# Book Reviews

**Published:** 1875-09

**Authors:** 


### Book Reviews.

[NOTE. — All works reviewed in the pages of the CHICAGO MEDICAL JOURNAL AND EXAMINER may be found in the extensive stock of W. B. KEEN, COOKE & Co., whose catalogue of Medical Books will be sent to any address upon request.]

1. PLAIN DIRECTIONS FOR ACCIDENTS, EMERGENCIES, AND POISONS. Keep this where you can readily find it. By a Fellow of the College of Physicians of Philadelphia, and Physician to several of the Charitable Institutions of the same city. Fiftieth thousand. All rights reserved by the Author. Distributed to its Policy-Holders by the Mutual Life Insurance Company of New York. F. S. Winston, President.

2. PLAIN DIRECTIONS FOR THE CARE OF THE SICK, AND RECIPES FOR SICK PEOPLE. Keep this where you can readily find it. By a Fellow of the College of Physicians of Philadelphia, and Physician to several of the Charitable Institutions of the same city. Eightieth thousand. All rights reserved by the Author. Distributed to its Policy-Holders by the Mutual Life Insurance Company of New York. F. S. Winston, President.

These two little volumes are unique. Their gaily-illuminated covers suggest the dime novel and the ladies' magazine, while their contents treat of the accident in the street and the mishap in the family.

We are of those who advocate the widest possible dissemination of useful knowledge among the masses. But we are also of opinion that it is possible to give a little information, in certain cases, which may prove to be a dangerous thing. There are physicians who, in the sick room, discourse in such a manner as to produce the impression that they are the sole repositories of an incommunicable science. There are others yet who, on every possible occasion, deliver a species of medical lecture to all who chance to surround the sick bed. They who are truly wise do neither. The volumes before us, while they contain many excellent suggestions which a skilful practitioner would endorse, yet share the error of the second class of men.

On page 48 of the first volume, for example, two unprofessional fingers are represented in the act of strapping an extensive incised wound of the thigh—such a wound as the veriest tyro in surgery would not attempt to close without the aid of sutures. If the artist intended to represent a temporary dressing merely, the procedure suggested is laughably inadequate. The strips of plaster are cut so short as to be entirely unfit for the purpose, and are as few and far between as the individuals who succeed in carrying the policy of an insurance company during the term of their natural lives. To speak more plainly, less than half of those who are insured are said to remain so; and there is a great deal less than half enough plaster on the aforesaid wound.

Still, a very little of the latter might go a long way, if the author can be believed; for he asserts, on the very next page, that "the edges of an incised wound of some length were successfully held together by a *postage stamp*, divided lengthwise into four strips"!

Here are also directions for removing foreign bodies from punctured wounds, by *making incisions* along the course of the wound; directions for *splitting the nail* with a penknife; directions regarding the treatment of *sloughing wounds*, with a description of the mode of repair by granulation. Here are also indications for using such remedies as aqua ammoniæ, tincture of opium, oil of turpentine, ipecac in powder and syrup, Glauber's salt, and compound cathartic pills.

We submit that if an author is disposed to write a book on Minor Surgery, it were well to indicate his purpose in its title. Show us one hundred policy-holders of the Mutual Life Insurance Company, who treat themselves for extensive incisions along the thigh, punctured wounds requiring interference, wounds that slough and wounds that granulate—who move their bowels with Glauber's salt, and empty their stomachs with sulphate of zinc; and we will point out a certain proportion of the entire number whose widows are likely to realize from the investments made for them in the Company!

These volumes of themselves would scarcely deserve attention, were it not for the fact that they are circulated by a wealthy corporation, commanding a capital of seventy-two millions of dollars, and having ninety thousand policy-holders. One of these monographs has reached an edition of eighty thousand copies—truly an enormous edition for any work!

The author says there is scarcely any excuse for the "effrontery" of ringing up an apothecary, of a cold night, to supply ten cents worth of paregoric. Will the Mutual Life Insurance Company of New York excuse the "effrontery" of our asking why, when it was concluded to publish such volumes for the benefit of their policy-holders, they felt constrained to step outside of the medical profession of their own city, in order to find an author in Philadelphia?

LESSONS ON PRESCRIPTIONS AND THE ART OF PRESCRIBING. By *W. Handel Griffiths, Ph.D., L.R.C.P.E.* London: Macmillan & Co. 1875. 12mo., pp. 150. Price, $1.25.

Its title indicates its design, which it well fulfills. By careful attention to its instructions, a novice can learn to write a prescription accurately, in a very short time. One point the author fails to emphasize, which should be insisted on with something akin to energy, viz., an avoidance of abbreviations in writing prescriptions. Chapter 6 is on "Incompatibility," and it is worth the price of the book. If it were in the reviewer's power, every graduate of medicine in this country would commit this chapter to memory. Nearly the first half of the book—65 pages—is didactic, and the remainder consists of "Examples and Exercises." The subject of prescription writing is so thoroughly written up, in works on Materia Medica, that nothing new remains to be culled by writers on this subject alone. This work embraces everything of profit to the senior student and practitioner, and will repay studying. E.

### BOOKS RECEIVED.

BASTIAN.—Paralysis from Brain Diseases, in its common forms. Illustrations. D. Appleton & Co. $1.75.

DARWIN.—Insectivorous Plants. With illustrations. D. Appleton & Co. $2.00.

PAGET.—Clinical Lectures and Essays. D. Appleton & Co. $5.00.

### PAMPHLETS RECEIVED.

ANALYSIS OF ONE THOUSAND CASES OF SKIN DISEASE, with Cases and Remarks on Treatment. By *L. Duncan Bulkley, A.M., M.D.*, Physician to the Skin Department, Demilt Dispensary, N. Y. Reprinted from The American Practitioner, May, 1875. Louisville: J. P. Marten & Co.

AMERICAN CLINICAL LECTURES, Vol. 1, No. vii; Capillary Bronchitis of Adults. By *Calvin Ellis, M.D.*, Prof. of Clinical Medicine, Harvard University. New York: G. P. Putnam & Sons.

ANNUAL ADDRESS before the California State Medical Society. By *A. B. Nixon, M.D.* Sacramento: Printed by H. S. Crocker & Co.

ANNUAL ANNOUNCEMENTS for 1875–6, from following Medical Schools: Atlanta Medical College; Woman's Hospital Medical College, of Chicago; Savannah Medical College; Department of Medicine and Surgery, University of Nashville; Second Annual Announcement of the Hospital College of Medicine, Louisville, Ky., Medical Department of Central University; National Medical College of The Columbian University; University of Michigan, Department of Medicine and Surgery.

### JOURNALS RECEIVED.

The American Journal of the Medical Sciences—1875. August.
The American Journal of Pharmacy—1875. August.
The American Medical Weekly—1875. August 7, 14, and 21.
The American Practitioner—1875. August.
The Atlanta Medical and Surgical Journal—1875. August.
The Archives of Dermatology—1875. July.
The Boston Medical and Surgical Journal—1875. August 5, 12, and 19.
The Canada Lancet—1875. August.
The Canada Medical and Surgical Journal—1875. August.
The Cincinnati Lancet and Observer—1875. August.
The Cincinnati Medical Advance—1875. August.
The Clinic—1875. August 7, 14, and 21.
The Dental Cosmos—1875. August.
The Detroit Review of Medicine and Pharmacy—1875. August.

The Druggists' Circular—1875. August.
The Eclectic Medical Journal—1875. August.
The Eclectic Medical Journal of Pennsylvania—1875. August.
The Herald of Health—1875. August.
The Journal of Materia Medica—1875. August.
The London Lancet—1875. August.
The Medical News and Library—1875. August.
The Medical Record, New York—1875. August 7, 14, and 21.
The Medical and Surgical Reporter, Philadelphia—1875. August 7, 14, and 21.
The Monthly Abstract of Medical Sciences—1875. August.
The Nashville Journal of Medicine and Surgery—1875. August.
The New York Medical Journal—1875. August.
The Pacific Medical and Surgical Journal—1875. August.
The Peninsular Journal of Medicine—1875. August.
The Philadelphia Medical Times—1875. August 7, 14, and 21.
The Sanitarian—1875. August.
The Sanitary Journal—1875. August.
The St. Louis Medical and Surgical Journal—1875. August.
The United States Medical Investigator—1875. August 2.
Der Feldartz—1875. Nr. 12.
Der Irrenfreund—Jahr. xvii, Nr. 4.
L'Anatomie et de la Physiologie Journal de. Ch. Robin—No. 4, Mai et Juillet & Soût, Paris, 1875.
La France Medicale—1875. Nrs. 53, 54.
Le Progres Medicale, Paris, 3e Annee—Nos. 26, 27, 28, 29, 30.
Memorabilien—Jahr. xx, Heft. 4.

---

Dr. Lyman Ware, just returned from Europe, informs us that graduates in medicine who may be going abroad to study, will be admitted—on showing their diplomas, and the payment of a small fee—to six months dressing and perpetual medical and surgical practice, to the London Hospitals, which is unequaled in the way of many practical advantages.

